# Relevance of RNA N6-Methyladenosine Regulators for Pulmonary Fibrosis: Implications for Chronic Hypersensitivity Pneumonitis and Idiopathic Pulmonary Fibrosis

**DOI:** 10.3389/fgene.2022.939175

**Published:** 2022-07-14

**Authors:** Yiyi Zhou, Chen Fang, Qinying Sun, Yuchao Dong

**Affiliations:** Department of Respiratory and Critical Care Medicine, Changhai Hospital, Second Military Medical University, Shanghai, China

**Keywords:** chronic hypersensitivity pneumonitis, idiopathic pulmonary fibrosis, m6A, immune microenvironment, consensus clustering

## Abstract

N6-methyladenosine (m6A) modification plays a pivotal role in post-transcriptionally regulating gene expression and biological functions. Nonetheless, the roles of m6A modification in the regulation of chronic hypersensitivity pneumonitis (CHP) and idiopathic pulmonary fibrosis (IPF) remain unclear. Twenty-two significant m6A regulators were selected from differential gene analysis between the control and treatment groups from the GSE150910 dataset. Five candidate m6A regulators (insulin-like growth factor binding protein 2, insulin-like growth factor binding protein 3, YTH domain-containing protein 1, zinc finger CCCH domain-containing protein 13, and methyltransferase-like 3) were screened by the application of a random forest model and nomogram model to predict risks of pulmonary fibrosis. The consensus clustering method was applied to divide the treatment samples into two groups with different m6A patterns (clusters A and B) based on the 22 m6A regulators. Our study performed principal component analysis to obtain the m6A-related score of the 288 samples to quantify the two m6A patterns. The study reveals that cluster A was linked to T helper cell (Th) 2-type cytokines, while the immune infiltration of Th1 cytokines was higher in cluster B. Our results suggest that m6A cluster A is likely related to pulmonary fibrosis, indicating m6A regulators play notable roles in the occurrence of pulmonary fibrosis. The m6A patterns could be considered as biomarkers to identify CHP and IPF, which will be helpful to develop immunotherapy strategies for pulmonary fibrosis in the future.

## Introduction

As a form of hypersensitivity pneumonitis (HP), chronic hypersensitivity pneumonitis (CHP) is an inflammatory and/or fibrotic disease affecting the lung parenchyma and small airways. It typically results from an immune-mediated reaction provoked by an overt or occult inhaled antigen in susceptible individuals (Koster et al., 2021) and carries the most significant morbidity and mortality (Campbell, 1932; Morell et al., 2013). Though high prevalence occurs in older individuals (Invernizzi et al., 2021), it can also hit younger age groups (Hanak et al., 2007; Morell et al., 2008). Common symptoms include dyspnea, chronic dry cough, chest tightness, flu-like symptoms, mid-inspiratory squeaks (Koster et al., 2021), and IPF (Lederer and Martinez, 2018). Approximately 11%–65% of patients with CHP have chest imaging features of pulmonary fibrosis (Vourlekis et al., 2004; Sahin et al., 2007; Hanak et al., 2008; Fernández Pérez et al., 2013; Mooney et al., 2013; Adegunsoye et al., 2016; Johannson et al., 2016; Chung et al., 2017) and appear to be clinically similar to subjects with IPF. Yet now, those categories (acute, subacute, or chronic) of HP were vaguely defined in current literature (Lacasse et al., 2003). Furthermore, a previous study indicated that diagnostic types and outcomes are inconsistent (Hanak et al., 2007). The histopathologic patterns in CHP include one type named usual interstitial pneumonia (UIP), with a course similar to idiopathic pulmonary fibrosis (IPF), which results in clinically indistinguishable disease and delays the identification and avoidance of the CHP inciting antigens (Furusawa et al., 2020). On review, UIP/IPF is being over-diagnosed and many cases so labeled is actually CHP with a UIP-like pattern (Churg et al., 2018). The pathogenesis of IPF posits that repeated subclinical epithelial cell injury superimposed on accelerated epithelial aging leads to aberrant repair of damaged alveoli and deposition of interstitial fibrosis induced by myofibroblasts (Lederer and Martinez, 2018). However, the molecular relationship between CHP and IPF is not comprehended. Hence, early screening of high-risk crowds and distinguishing CHP from IPF will profoundly impact the effective treatment of the two diseases.

In the 1960s, scientists discovered modified nucleotides in abundant cellular RNAs (Cohn, 1960). RNA modifications, including N6-methyladenosine (m6A), uridylation (U-tail), 5-methylcytidine (m5C), and N1-methyladenosine (m1A) have been identified in a great variety of RNA molecules with novel technology (Adams and Cory, 1975; Lee et al., 2014; Roundtree et al., 2017; Xu et al., 2021). For example, research on ovarian cancer (OC) patients suggested that the high m1A score group showed marked therapeutic benefits and clinical outcomes of chemotherapy and immunotherapy (Liu et al., 2021). RNA-modifying proteins (RMPs) usually participate in the progression of RNA methylation, which are called “writers,” “readers,” and “erasers” (Frye et al., 2018). Among the different RNA modifications, m6A remains the best characterized at the functional level and the most abundant modifications in mRNA (Zaccara et al., 2019). METTL3, the first identified gene in m6A modification, can interact with Per2 and Arntl to directly mediate the export of mRNA (Bhattarai et al., 2021). METTL14, METTL16, WTAP, VIRMA, ZC3H13, CBLL1, RBM15, and RBM15B are also included in the complex of m6A “writers” (Meyer and Jaffrey, 2017; Wen et al., 2018; Gu et al., 2022; Shen et al., 2022; Su et al., 2022). Accordingly, YT521-B homology (YTH) domain family is the main “readers” (Jones et al., 2022) and “erasers” consisting of FTO and ALKBH5 (Kaur et al., 2022). Recent studies have focused on the importance of m6A modification in tumors (Jiang et al., 2022; Ma et al., 2022; Xiong et al., 2022; Yadav et al., 2022) and non-neoplastic diseases like asthma and pulmonary hypertension (Teng et al., 2021; Zeng et al., 2021; Zhou et al., 2021). Nevertheless, the expression pattern of m6A regulators in CHP and IPF remains unclear.

In our study, RNA-seq data of human lung tissues were isolated from participants as either a surgical lung biopsy or at the time of lung transplant. We evaluated the functions of m6A regulators in diagnosis and classification of chronic hypersensitivity pneumonitis and idiopathic pulmonary fibrosis based on the GSE150910 dataset. More importantly, we uncovered the m6A regulator-mediated RNA methylation modification patterns and immune microenvironment infiltration characterization, which will be helpful to the diagnosis and treatment of these two diseases.

## Methodology

### Data Collection and Processing

The study incorporated GSE150910 containing 103 unaffected controls, 103 patients with IPF, and 82 patients with CHP from the NCBI gene expression synthesis database (NCBI–GEO, https://www.ncbi.nlm.nih.gov/geo). Using R package “biomaRt”, the whole data set was filtered and validated by deleting missing and duplicated data and transformed by using statistical procedure log2 (TPM). 25 m6A regulators were extracted using “limma” from R package. We focused on a total of 8 writers (METTL3, METTL14, METTL16, WTAP, VIRMA, ZC3H13, RBM15, and CBLL1), 15 readers (YTHDC1, YTHDC2, YTHDF1, YTHDF2, YTHDF3, HNRNPC, FMR1, LRPPRC, HNRNPA2B1, IGFBP1, IGFBP2, IGFBP3, RBMX, ELAVL1, and IGF2BP1), and 2 erasers (FTO and ALKBH5). METTL16, LRPPRC, and FTO were removed from the list because they did not change significantly from differential expression analysis between patients (CHP, IPF) and controls.

### Development of the RF Model, SVM Model, and Nomogram Model

We systematically constructed two machine learning classifiers, random forest (RF) and support vector machine (SVM) to predict the occurrence of CHP and IPF. Random forests are a combination of tree predictors, which mitigates individual biases by combining and weighting the regression or classification (Breiman, 2001; Chen et al., 2022).

We established an RF model to select candidate m6A regulators among the 22 m6A regulators. Then, the importance of the 22 m6A regulators was checked and five regulators with the highest score were selected. When it comes to algorithm regarding SVM classification, four features (the separating hyperplane, the maximum-margin hyperplane, the soft margin, and the kernel function) need to be captured (Schölkopf and Smola, 2002; Noble, 2006). Every data point was plotted as a dot in n-dimensional spaces (Dai et al., 2021) in this research, and a hyperplane distinguishing control and treatment group (CHP and IPF) was identified. Subsequently, we plotted “boxplots,” “reverse cumulative distribution” and “receiver operating characteristic (ROC) curves” to evaluate the model. We established a nomogram prognostic model based on the 5 selected m6A regulators to predict the prevalence of CHP and IPF. Statistical analyses were performed using R packages: “rms” and “rmda”. The “calibration curve,” “clinical impact curve” and “decision curve analysis (DCA)” were plotted to evaluate the accuracy of the model and assess its validity.

### Consensus Clustering Analysis and Principal Component Analysis

To determine whether m6A regulators are associated with CHP and IPF, the cohort from GEO was divided into different groups based on the consensus level of m6A regulators, and the analysis was performed using R package “ConsensusClusterPlus”. The graphical output consisted of consensus cumulative distribution function (CDF) plots, delta area plots, and heatmaps. Principal component analysis (PCA) was utilized to determine the fitness of the classification.

### Differential Gene Expression Analysis and Gene Ontology Functional Enrichment Analysis of m6A Regulators

The “limma” package was conducted to identify the differentially expressed genes (DEGs) associated with m6A modifications. adjusted *p* value < 0.05 and absolute value of logFC>1 were used as the cutoff point. An R package “clusterProfiler” was used to analyze Gene Ontology (GO) functional enrichment. The significance criteria for GO analysis were set as an adjusted *p* value < 0.05.

### Immune Infiltrate Analysis

Single sample gene set enrichment analysis (ssGSEA) is an extension of GSEA and an implementation method proposed for single sample. The analysis calculates separate enrichment scores for each pair of a sample and gene set (Barbie et al., 2009; Liu et al., 2020a). We used ssGSEA to calculate the abundance of immune cells in treatment groups. Next, Immuno-correlation analysis was carried out based on ssGSEA and the results were visualized using heatmaps and boxplots.

### Weighted Gene Co-Expression Network Analysis

The modification pattern-related genes and gene modules were identified by weighted gene co-expression network analysis (WGCNA) using the R package “WGCNA”. A heatmap was plotted to explore the relevance among genes, modules, and types of samples. Finally, kyoto encyclopedia of genes and genomes (KEGG) analysis based on gene modules was conducted. The significance criteria were set as adjusted *p*-value less than 0.05.

### Statistical Analysis

The correlation between writers and erasers was analyzed using linear regression analyses. Wilcox-test was used to detect differences between several groups. Pairwise prop test was used to compare the differences of CHP and IPF groups. The efficiency of models was assessed through the area under the curve (AUC) using R package “pROC”. All data processing was done in R 4.1.2 software.

## Results

### Landscape of the m6A Regulators in Chronic Hypersensitivity Pneumonitis and Idiopathic Pulmonary Fibrosis

**FIGURE 1:**
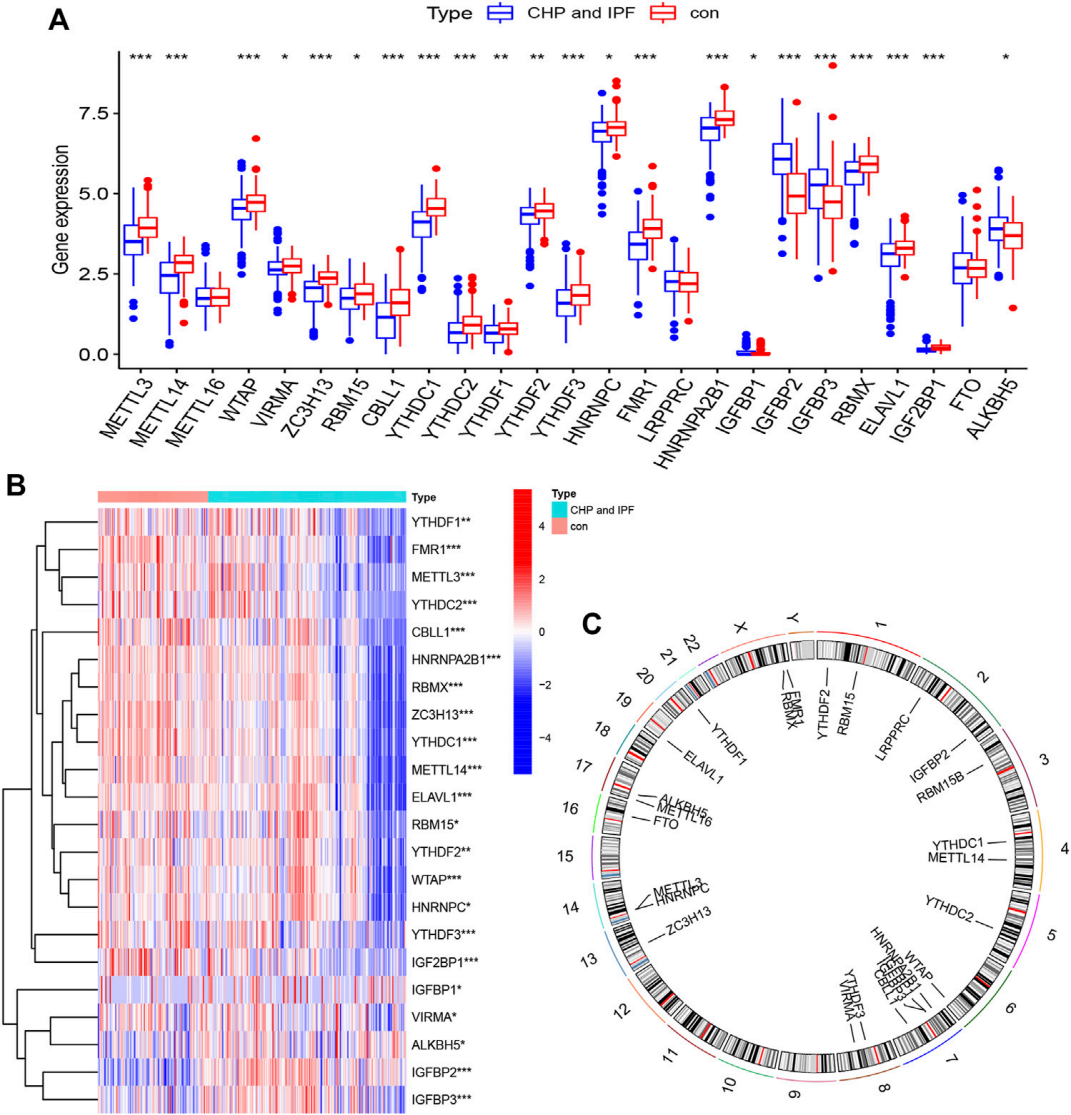
Landscape of the N6-methyladenosine (m6A) regulators in CHP and IPF **(A)** The differential expression histogram of 25 m6A regulators between controls and tests. **(B)** The differential expression heatmap of 22 m6A regulators. **(C)** Chromosomal positions were visualized using the “RCircos” package. *p < 0.05, **p < 0.01, and ***p < 0.001.

The m6A modification is a reversible biological process, mainly consisting of three related enzymes (Liao et al., 2022). Writers, RNA m6A methyltransferase complex, identify the target RNA and accelerate cytoplasmic export as well as protein translation (Lan et al., 2021). Only two erasers, which exist as demethylase, can identify m6A modification sites and reverse m6A to A (Vu et al., 2019a). Readers bind m6A-modified RNAs and mediate its realization function, including splicing (Kasowitz et al., 2018), mRNA stability (Liu et al., 2020b), mRNA translation (Shi et al., 2017), and so on. A total of 25 m6A regulators including 8 writers, 2 erasers, and 15 readers were investigated in this study. R package “limma” was utilized to locate m6A regulators with significant variation, and the differential expression levels of 25 m6A regulators between controls and tests (CHP and IPF) were presented (Figure 1A). Figure 1B was same as Figure 1A, which was a presentation of the expression levels between controls and tests. Subtracting one from each of writers, erasers, and readers, 22 significant m6A regulators (METTL3, METTL14, WTAP, VIRMA, ZC3H13, RBM15, CBLL1, YTHDC1, YTHDC2, YTHDF1, YTHDF2, YTHDF3, HNRNPC, FMR1, HNRNPA2B1, IGFBP1, IGFBP2, IGFBP3, RBMX, ELAVL1, IGF2BP1, and ALKBH5) were screened and visualized (Figure 1B). Among them, IGFBP2, IGFBP3, and ALKBH5 were markedly overexpressed in the tests and decreased expression in the controls. Chromosomal positions of the m6A regulators were shown in Figure 1C.

### Correlation Between Writers and Erasers

**FIGURE 2:**
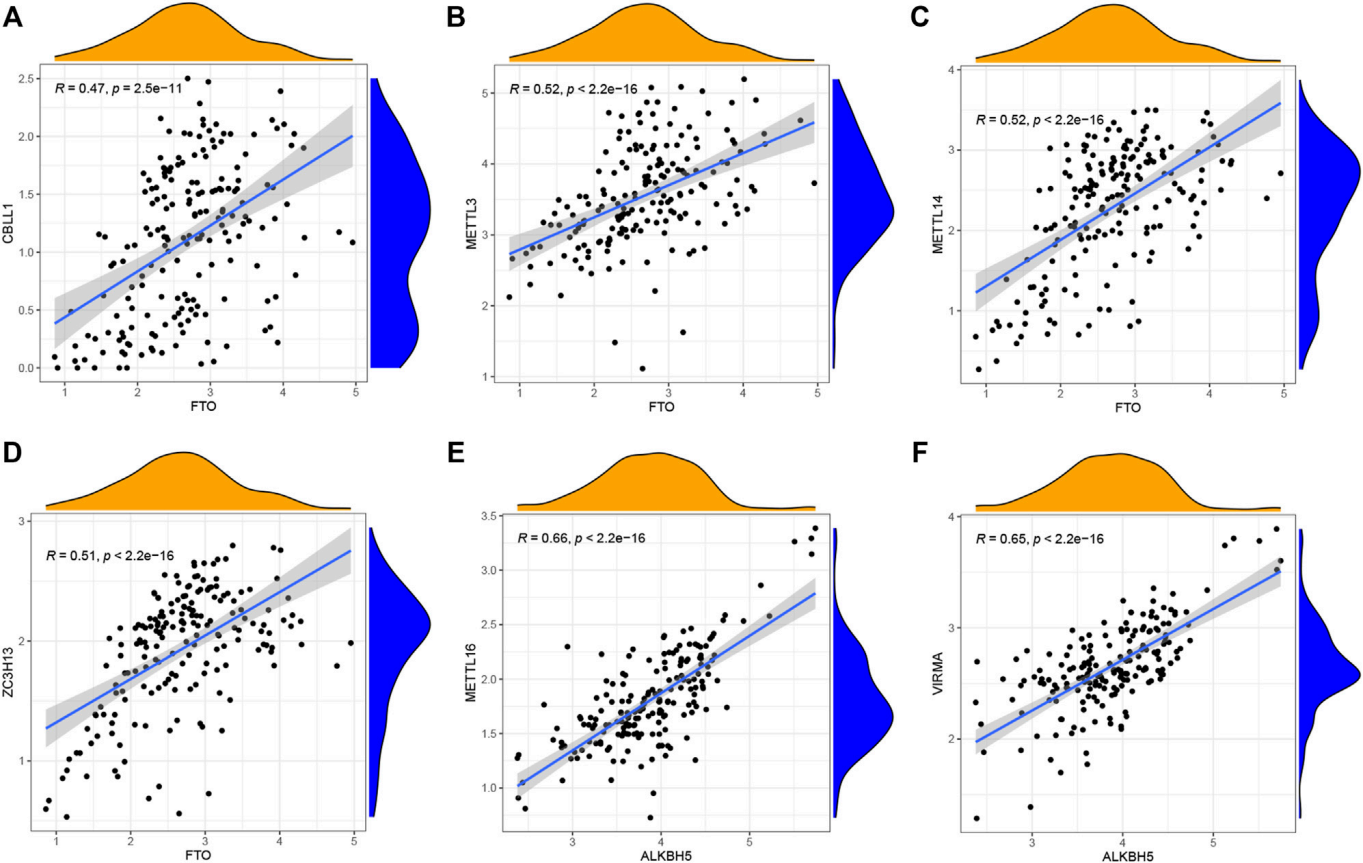
Correlation between writers and erasers in CHP and IPF **(A–F)** Writer genes: CBLL1, METTL3, METTL14, ZC3H13, METTL16, and VIRMA. Eraser genes: FTO and ALKBH5 (|R| > 0.4 and *p* < 0.001). R, correlation coefficient.

Linear regression analyses were utilized to explore the correlation between m6A writers and erasers. The expression levels of four writers (CBLL1, METTL3, METTL14, and ZC3H13) showed high positive correlations with FTO (Figures 2A–D), while high levels of METTL16 and VIRMA expressions displayed positive correlations with ALKBH5 (Figures 2E,F). Supplementary Table S1 was provided for more details.

### Construction of the RF Model, SVM Model, and Nomogram Model

**FIGURE 3:**
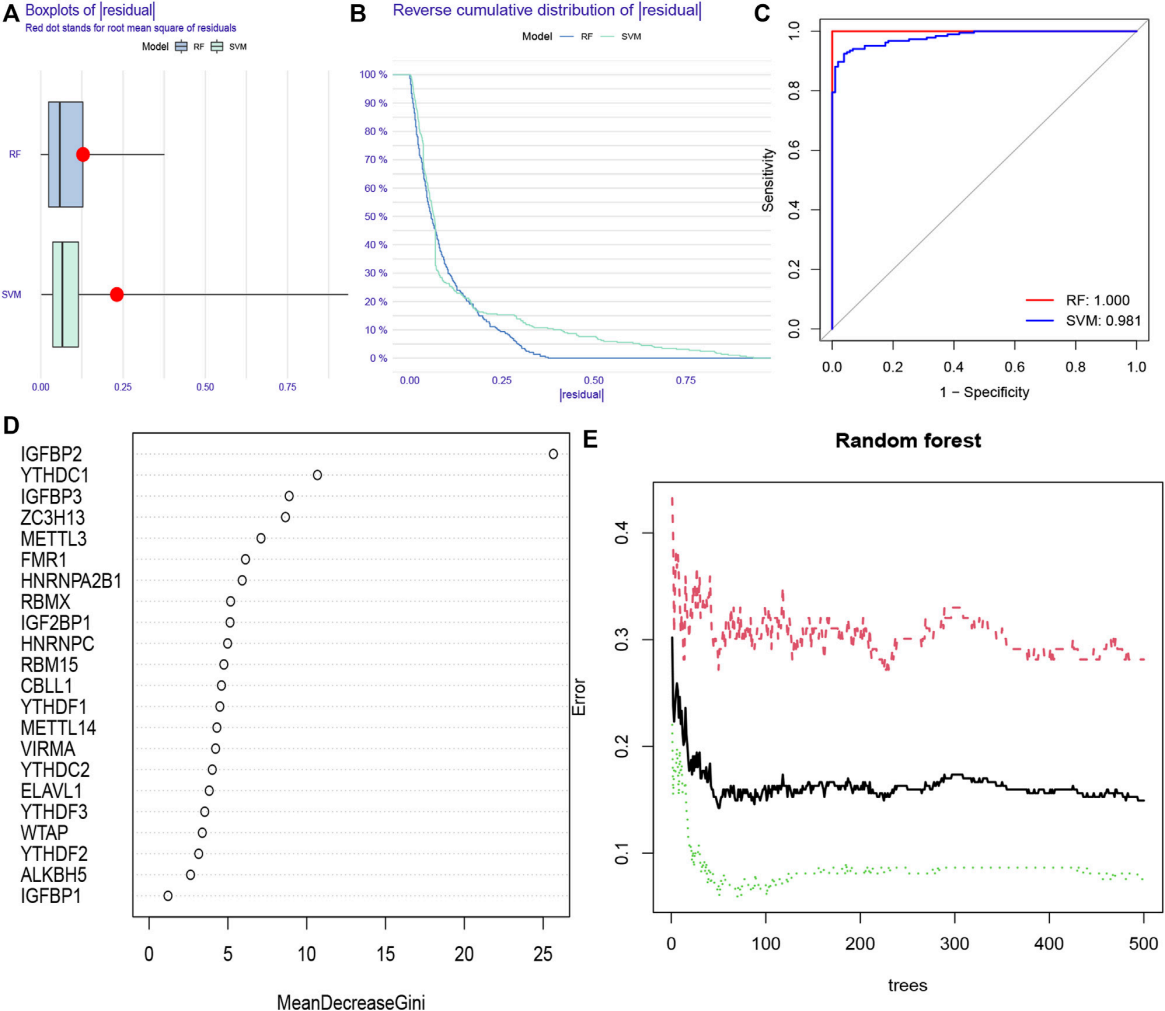
Random forest (RF) model and support vector machine (SVM) model construction **(A)** RF model showed a lower residual value in the boxplots of residual. **(B)** Reverse cumulative distribution of residual was plotted to show the residual distribution of RF and SVM model **(C)** RF model and SVM model displayed a favorable predictive value, with an AUC of 0.981 for SVM, and 1.000 for RF. **(D)** The importance of the 22 RNAN6-methyladenosine regulators was calculated based on the RF model, including the top five regulators (IGFBP2, YTHDC1, IGFBP3, ZC3H13, and METTL3) **(E)** The three curves represented the error levels of treat groups (red line), control groups (green line), and overall samples (black line), respectively.

RF and SVM models were simultaneously established to screen for m6A regulators which could characterize diseases. Both of them were meaningful for the prediction of occurrence. We found that the RF model was more appropriate than SVM model using “Boxplots of residual,” “Reverse cumulative distribution of residual” and “ROC curve” (Figures 3A–C). Further analysis of selecting important m6A regulators was conducted using “randomForest” package. We searched the point where the cross-validation error was minimal and determined the importance of each feature (Figures 3D,E). The top five m6A regulators (IGFBP2, YTHDC1, IGFBP3, ZC3H13, and METTL3) based on the importance score were used for establishing a nomogram model to predict the prevalence of CHP and IPF patients.

**FIGURE 4:**
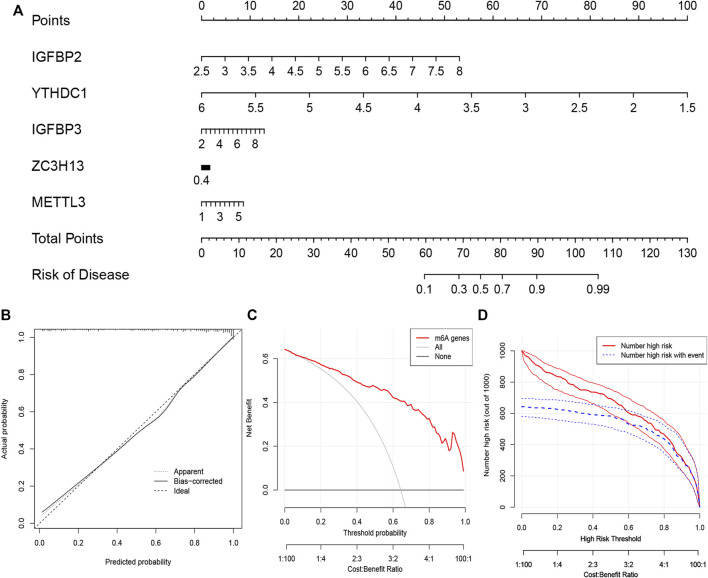
The nomogram model construction **(A)** The prevalence was predicted according to gene score. Total points of 60 represented a prevalence of 10%, while 90 scores meant a prevalence of 90%. **(B)** Calibration curve was constructed to assess the accuracy of nomogram model **(C)** The construction of the decision curve. **(D)** Clinical impact curve based on the five candidate RNA N6-methyladenosine regulators.

We constructed “nomogram,” “calibration curve,” “decision curve,” and “clinical impact curve” to assess the veracity. The prevalence was predicted according to gene score (Figure 4A). Calibration curves showed a short distance between the solid and dotted lines, indicating the accuracy of nomogram model (Figure 4B). On the contrary, the red line represented m6A genes in this decision curve had a deviation from gray and black lines (Figure 4C), verifying the conclusion. Similarly, the clinical impact curve revealed that the model was remarkable in Figure 4D.

### Two m6A Patterns Identified by Significant m6A Regulators

**FIGURE 5:**
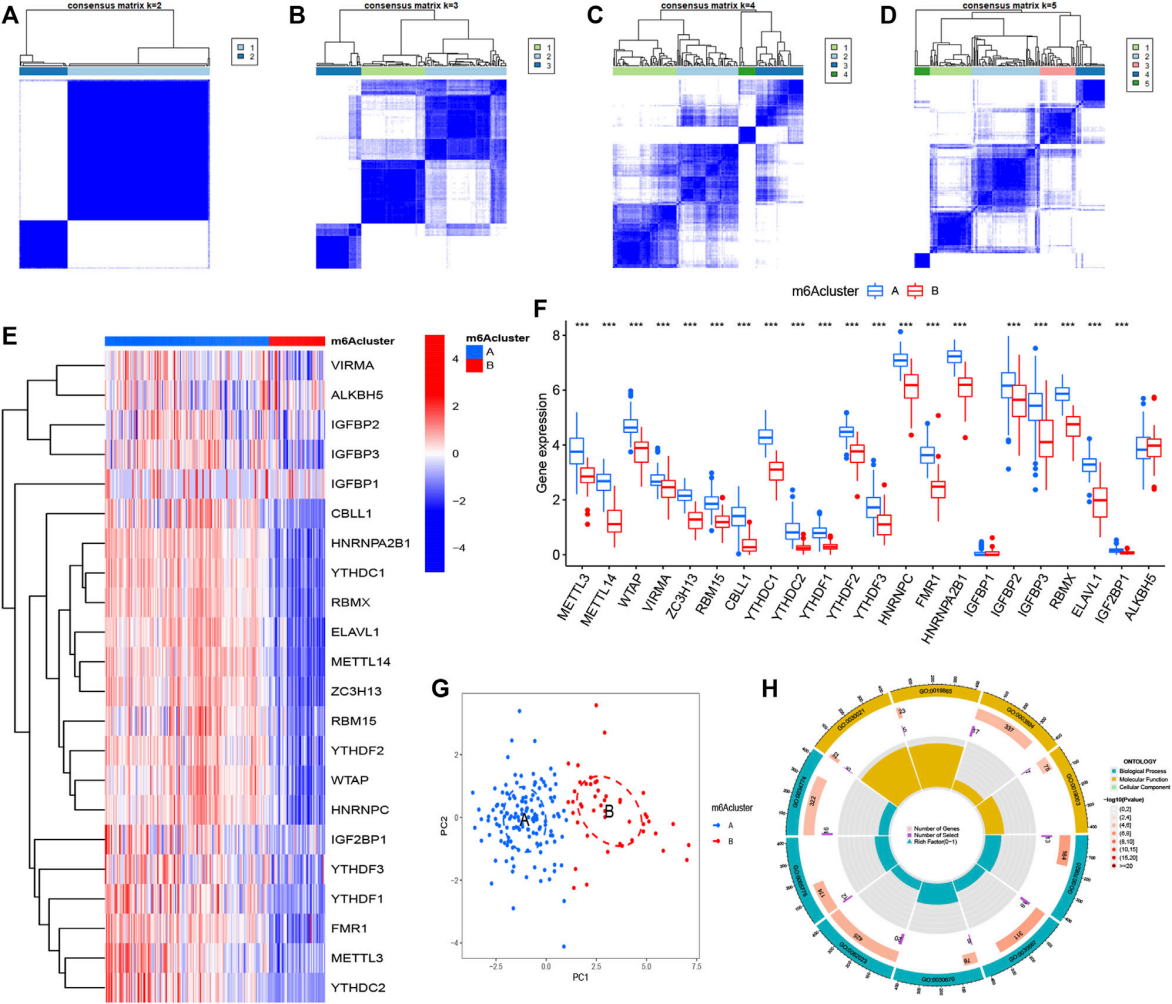
Consensus clustering of N6-methyladenosine (m6A) regulators in treat group (CHP and IPF) **(A–D)** Consensus matrices of the 22 significant m6A regulators (k = 2–5) **(E,F)** Expression heatmap and histogram of the 22 significant m6A regulators in cluster A and cluster B **(G)** Principal component analysis for the expression profiles of m6A subtypes, showing a remarkable difference between different modification patterns **(H)** The GO enrichment analysis for the 361 m6A-related differentially expressed genes (DEGs) uncovered potential mechanisms for the occurrence and development of the treatment group. *p < 0.05, **p < 0.01, and ***p < 0.001.

Our study selected consensus clustering method to seek distinct m6A patterns using R package “ConsensusClusterPlus” and the tests (CHP and IPF) were divided into m6A cluster A and cluster B (Supplementary Table S2) based on the variance analysis result file (Supplementary Table S3) obtained in Figure 1B. Results clearly showed that the m6A regulators divided into two groups (cluster number = 2) had a different pattern (Figures 5A–D). The heatmap and histogram based on cluster A and cluster B were plotted to demonstrate the differential expression levels of the 20 significant m6A regulators. METTL3, METTL14, WTAP, VIRMA, ZC3H13, RBM15, CBLL1, YTHDC1, YTHDC2, YTHDF1, YTHDF2, YTHDF3, HNRNPC, FMR1, HNRNPA2B1, IGFBP2, IGFBP3, RBMX, ELAVL1, and IGF2BP1 had higher expression levels in cluster A. Two m6A regulators (IGFBP1 and ALKBH5) illustrated no significant difference (Figures 5E,F). PCA data had shown that the 20 m6A regulators could clearly distinguish cluster A and cluster B (Figure 5G).

A total of 3,168 m6A-related DEGs were identified between cluster A and cluster B using the “limma” package, and GO functional enrichment analysis was conducted on the top 361 DEGs to explore underlying mechanisms between CHP and IPF (Figure 5H and Table 1). We found that the genes were mainly enriched in GTPase activity (GO:0003924), secretory granule lumen (GO:0034774), collagen-containing extracellular matrix (GO:0062023), and secretory granule membrane pathways (GO:0030667).

**TABLE 1:** Ten GO pathway names based on the 361 m6A-related DEGs.

GO Terms	Names
GO:0030021	extracellular matrix structural constituent conferring compression resistance
GO:0019865	immunoglobulin binding
GO:0003924	GTPase activity
GO:0019003	GDP binding
GO:0070820	tertiary granule
GO:0030667	secretory granule membrane
GO:0030670	phagocytic vesicle membrane
GO:0062023	collagen-containing extracellular matrix
GO:0005775	vacuolar lumen
GO:0034774	secretory granule lumen

GO, Gene Ontology; DEGs, differentially expressed genes.

**FIGURE 6:**
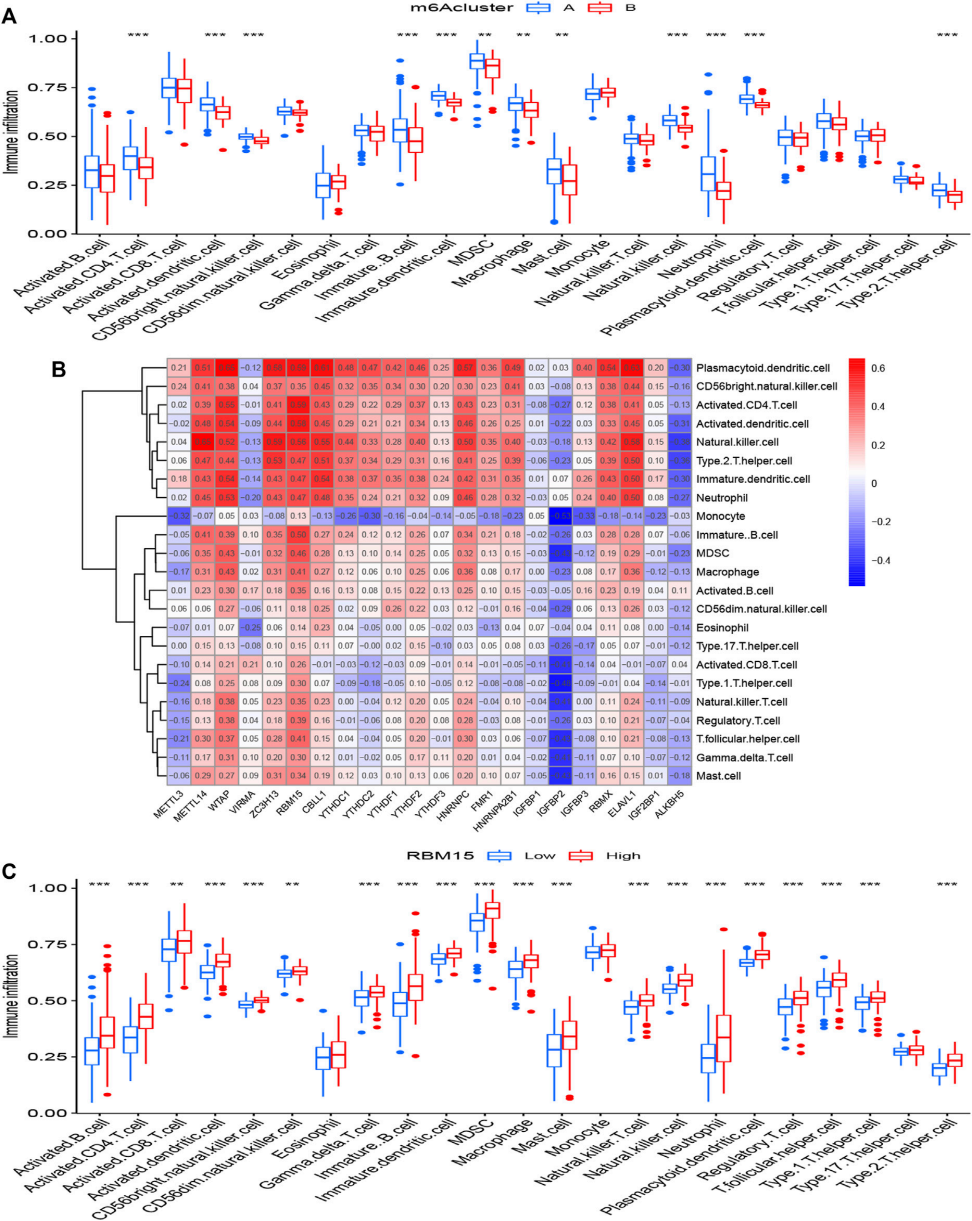
Single sample gene set enrichment analysis **(A)** The expression status of 22 m6A regulators in the two m6A patterns. **(B)** Correlation between the 22 significant RNA N6-methyladenosine regulators and infiltrating immune cells. **(C)** Differential immune cell infiltration between low RBM15 expression group and high RBM15 expression group. *p < 0.05, **p < 0.01, and ***p < 0.001.

The abundance of immune cells in treatment samples was calculated by ssGSEA. We explored the differential immune cell infiltration between cluster A and cluster B. Our data showed that cluster A was linked to Th2 cytokines (*p* < 0.001). Though the data detected no significant difference between cluster B and Th1 cytokines (*p* > 0.05), the immune infiltration of Th1 cytokines in cluster B was higher than that in cluster A (Figure 6A). This result suggested that m6A cluster A may be related to pulmonary fibrosis. RBM15 had the most significant association with immune cells according to the Supplementary Table S4. Correlational analysis between RBM15 and immune cells was conducted (Figure 6B). In addition. compared to patients with low RBM15 expression, patients with high RBM15 expression had increased immune cell infiltration (Figure 6C).

### Identification of Two Distinct m6A Gene Patterns and Generation of the m6A Gene Signature

**FIGURE 7:**
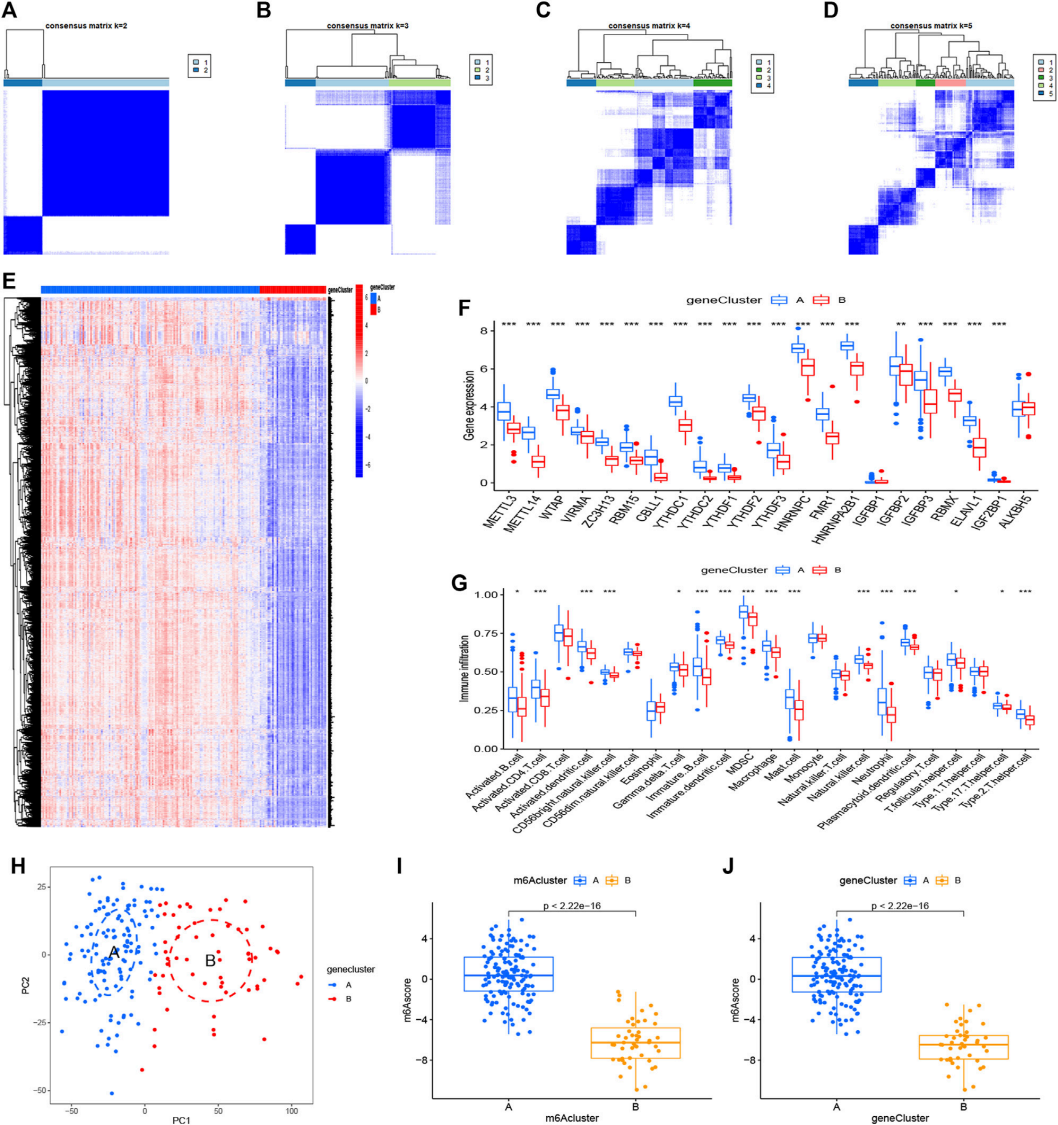
Consensus clustering of the 3,168 m6A-related DEGs **(A–D)** Consensus matrices of the DGEs (k = 2–5) **(E,F)** Expression heatmap and histogram of the 3,168 m6A-related DEGs in gene cluster A and gene cluster B **(G)** The differential immune cell infiltration of DEGs in the two gene patterns **(H)** Principal component analysis for the expression profiles of gene subtypes, also shows a remarkable difference between different modification patterns. **(I,J)** Differences in m6A score based on PCA algorithm between the two m6A patterns or the two gene patterns. *p < 0.05, **p < 0.01, and ***p < 0.001.

**FIGURE 8:**
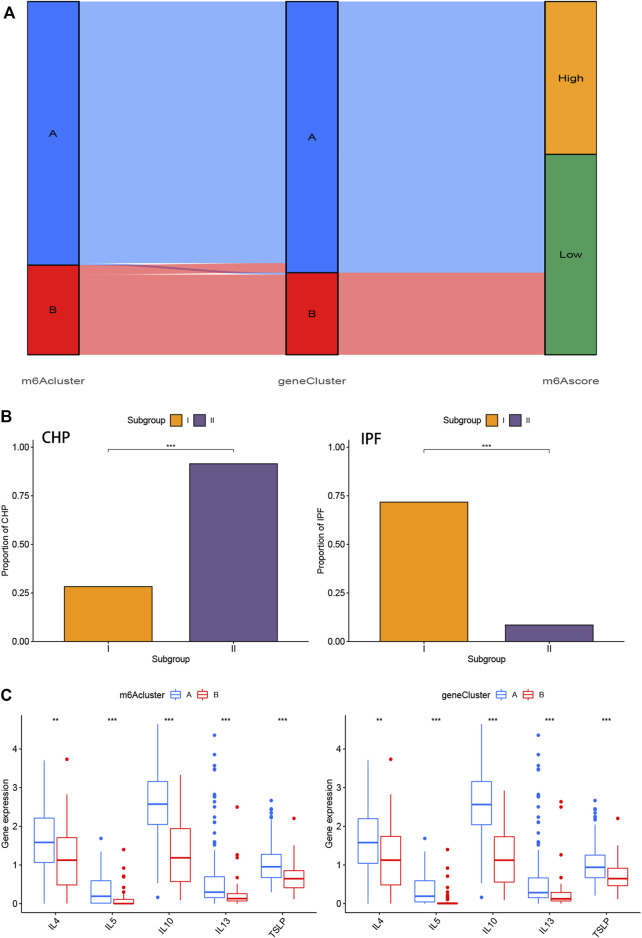
Role of m6A patterns and gene patterns in distinguishing pulmonary fibrosis **(A)** Sankey diagram of the relationship between two m6A patterns, two gene patterns, and m6A scores. **(B)** Correlation between m6A patterns and clinical types. The samples were divided into 2 groups (Subgroup I and II) based on m6A patterns, one for each of the m6Acluster A and m6Acluster B. The distribution of CHP in two m6A patterns was shown on the left, while the distribution of IPF was shown on the right **(C)** Differential expression levels of interleukin (IL)-4, IL-5, IL-10, IL-13 and thymic stromal lymphopoietin (TSLP) between m6Acluster A and m6Acluster B or gene cluster A and gene cluster B. *p < 0.05, **p < 0.01, and ***p < 0.001.

We utilized consensus clustering method to divide the treatment samples into different subtypes based on the 3,168 m6A-related DEGs. The result of gene patterns (cluster A and B) was found approximately parallel to the grouping of m6A patterns (m6A cluster A and m6A cluster B) (Figures 7A–D), which increased the credibility of m6A patterns. The heatmap based on the 3,168 m6A-related DEGs showed different expression levels between gene cluster A and gene cluster B (Figure 7E). Similar to the boxplots of m6A patterns, 12 m6A regulators had higher expression levels in gene cluster A and two regulators illustrated no significant differences (Figure 7F). Meanwhile, the gene cluster A was linked to immune cell infiltration (Figure 7G). All of the analyses validate the accuracy of our gene typing by the consensus clustering method. Principal component analysis (PCA), a classical statistical technique, analyzes the covariance structure of multivariate data (Weingessel and Hornik, 2000). PCA data had shown a remarkable difference between different modification patterns (Figure 7H). In order to quantify the m6A patterns, our study performed this algorithm above to obtain m6A-related score of the 288 samples. The m6A-related score in gene cluster A and m6A cluster A were both significantly higher than that in cluster B (Figures 7I,J). This Sankey diagram showed the relationship between different patterns and m6A-related scores more distinctly (Figure 8A).

### Role of m6A Patterns and Gene Patterns in Distinguishing Chronic Hypersensitivity Pneumonitis and Idiopathic Pulmonary Fibrosis

We performed the correlation analysis between m6A patterns and clinical types using “rstatix” package. Samples of IPF in Subgroup II (m6Acluster B) were significantly less than that in Subgroup I (m6Acluster A), and samples of CHP in Subgroup II was more than that in Subgroup I (Figure 8B). The results indicated that the different m6A patterns may distinguish CHP and IPF, more precisely, pulmonary fibrosis and non-fibrosis. The correlation between different patterns and Th2 cytokines (TSLP, IL-4, IL-5, IL-10, and IL-13) was exhibited in Figure 8C. We found that the expression levels of those cytokines were associated with remarkable differences in m6A Patterns or gene Patterns. Levels in cluster A were both higher than that in cluster, which indicated that m6Acluster A or gene cluster A was highly linked to pulmonary fibrosis mediated by Th2 cytokines.

### Biological Characteristics of Different Patterns

**FIGURE 9:**
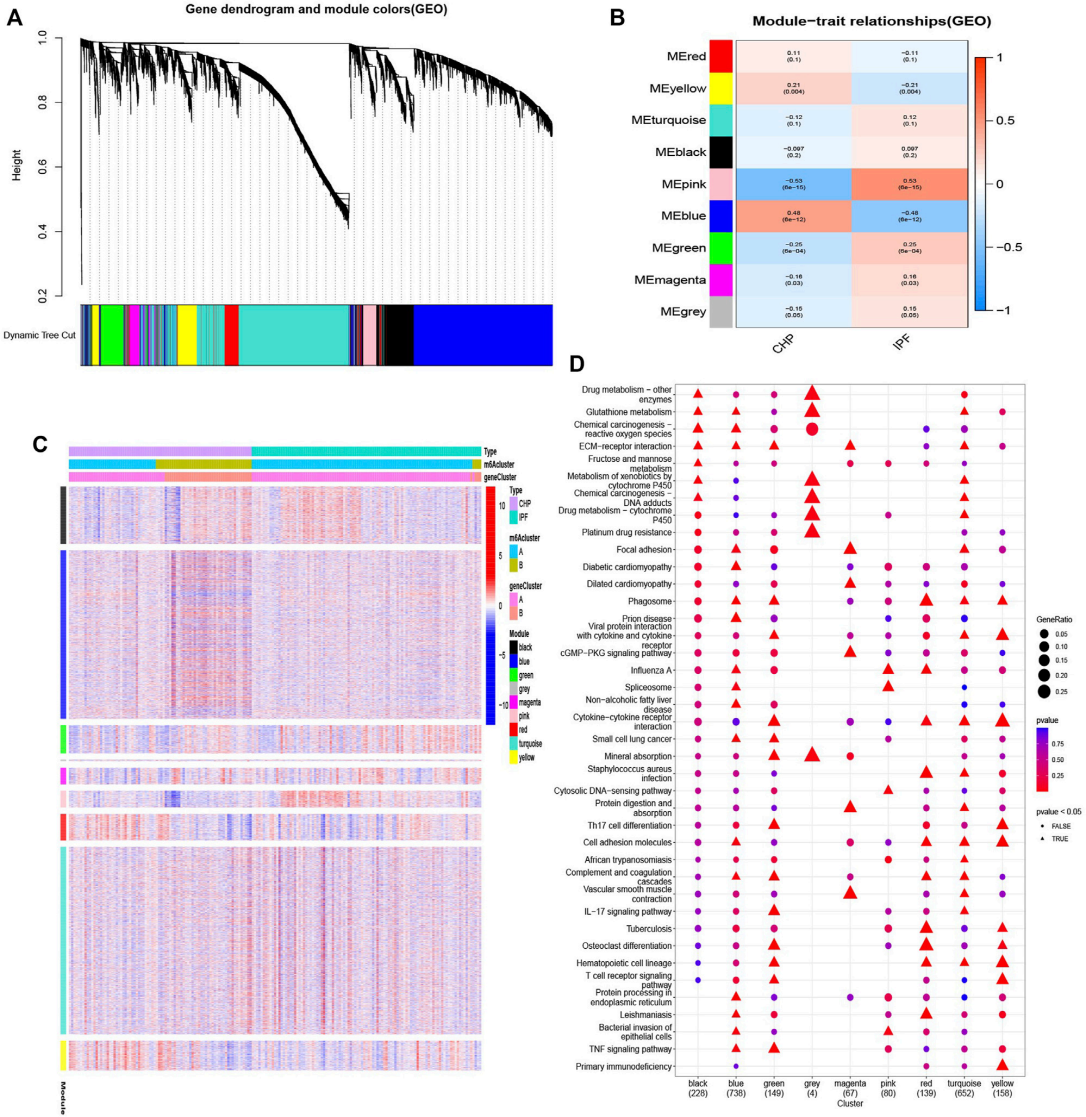
Biological characteristics of different patterns **(A,B)** Gene-gene modules related to different samples were identified by WGCNA. **(C)** Unsupervised clustering of overlapping m6A phenotype-related genes to classify patients into different clinical subtypes, termed as CHP and IPF, respectively. The gene clusters, m6Aclusters, and clinical subtypes were used as patient annotations. **(D)** Pathway annotations for each module using KEGG analysis. The color depth and graphic shape of the bubble represented the number of genes enriched. △ *p* < 0.05.

In order to reveal the regulatory models, we performed the WCGNA on the gene expression datasets. Nine gene modules were determined, and genes in blue modification pattern had the most significant difference (Figures 9A,B). To further explore the characteristics of the m6A modification phenotypes in the different clinical types and biological behaviors, we focused on different patterns constructed by m6A modification and conducted KEGG analysis based on gene-gene modules. Consistent with the above findings, most patients of CHP were clustered into m6Acluster B and almost no IPF subtypes were in m6Acluster B (Figure 9C). Moreover, the up-regulated genes in blue modification pattern were mainly enriched in small cell lung cancer, TNF signaling pathway, prion disease, diabetic cardiomyopathy, and ECM-receptor interaction pathways (Figure 9D).

## Discussion

Hypersensitivity pneumonitis is an interstitial lung disease (ILD), results from an immune-mediated response (Costabel et al., 2020). Unlike acute HP, chronic HP mostly originates from long-term and low-level exposure (Selman et al., 2012). It is difficult to have an unambiguous definition for CHP and part of researchers suggested that it is more significant to classify HP based on pulmonary fibrosis (Raghu et al., 2020). Imaging and clinical manifestations of CHP lead to frequent misdiagnosis, and idiopathic pulmonary fibrosis (IPF) is not excluded (Ohtani et al., 2003; Morell et al., 2013). In addition, when it comes to the therapies of ILDs, it is universally acknowledged that nonpharmacologic management strategies (smoking cessation, supplemental oxygen, pulmonary rehabilitation, and lung transplantations) and pharmacologic management strategies (pirfenidone and nintedanib) have reduced the rate of decline in pulmonary function (PF) in patients (Adegunsoye and Strek, 2016; Lederer and Martinez, 2018; Wells et al., 2020). The function of corticosteroids to speed up symptomatic and radiographic resolution of HP leads to corticosteroids and immunosuppressants, such as azathioprine, mycophenolate mofetil (MMF), and cyclophosphamide, being widely used as the treatment for CHP (Maher, 2020). A study of anti-fibrotic therapy in unclassifiable ILD (patients with the difficulty of discriminating CHP from IPF) demonstrated a significant effect of treatment on slowing progression of disease (Maher et al., 2020). Previous guidelines also recommended antacid therapy for IPF (Raghu et al., 2015). However, more clinical data suggested that it might increase a risk of respiratory infections (Kreuter et al., 2016). Recent studies indicated that the combination of prednisone and MMF was associated with a decreased incidence of adverse events and a reduction in prednisone dose (Adegunsoye et al., 2021). And increasing clinical trials highlight the importance of mesenchymal stem cells (MSC) for pulmonary fibrosis (Nayra Cardenes et al., 2016). Nevertheless, anti-fibrosis treatments still dominate throughout the treatment history. The function of the m6A regulators involved in CHP and IPF remains unclear, thus, we hypothesized that m6A regulators might have an underlying association with CHP or IPF, and explore a new approach to pulmonary fibrosis treatment.

Here, we found expression of the major m6A regulators altered between control group and treatment group, suggesting m6A regulators participated in the disease evolution of CHP and IPF. 22 significant m6A regulators were identified based on 288 samples using differential expression analysis. The five candidate m6A regulators (IGFBP2, YTHDC1, IGFBP3, ZC3H13, and METTL3) based on the RF model were utilized for the construction of nomogram model to predict the prevalence of patients with CHP or IPF. Multiple curves were built to assess the veracity of nomogram model, and our results indicate that the model may benefit the early diagnosis and prompt treatment of those patients.

Two types of T cells (Th1 and Th2) were distinct according to patterns of lymphokine activity production *in vitro* (Mosmann et al., 1986). Type 1 T helper cells (Th1) produce IFN-γ, IL-2, IL-12, IL-18, and TNF-β, whereas type 2 helper T cells (Th2) produced IL-3, IL-4, IL-5, IL-10, IL-13, and MCP-1. Many prior studies proved that Th2 pattern of cytokines predominates in fibrosis. Wallace et al. (Wallace et al., 1995) first proposed “superiority hypothesis of Th2”. Huaux F et al. (Huaux et al., 1998) found that increased IL-10 synthesis induced by silica can limit the amplitude of the inflammatory reaction and contribute to amplifying the response of lung fibrotic. IL-4 and IL-5 were also discovered in the promotion of pulmonary fibrosis (Gharaee-Kermani et al., 1998; Nelms et al., 1999). Instead, IFN-γ has an antifibrosis effect, with hyposecretion in IPF and the pulmonary fibrosis model of rats (Vu et al., 2019b; Maeyama et al., 2020). The Th2-dominant response arising from Th1/Th2 imbalance is regarded as one of the vital immunological mechanisms of pulmonary fibrosis. Based on the 22 m6A regulators above, we revealed two distinct m6A methylation modification patterns. The two m6A patterns had significantly distinct immune cell infiltration characterization. Cluster A was linked to Th2 cytokines, whereas cluster B was linked to Th1 cytokines. The results demonstrated that patients in m6A cluster A may have a higher possibility of lung fibrotic. Subsequently, we calculated the proportion of CHP and IPF samples in different patterns and found that patients with IPF in cluster A were significantly higher than in the other group. The two m6A patterns will be beneficial to distinguish between patients with fibrosis and non-fibrosis. Nevertheless, GSE150910 dataset lacked clinical data about fibrosis in patients with CHP, it was unclear whether CHP in m6Acluster A got pulmonary fibrosis. On the other side, the diagnosis of CHP is ambiguous through imaging and clinical manifestations, given the absence of identifiable exposure as high as 50% of patients with fibrotic HP (Koster et al., 2021). Pathology and inter-multidisciplinary team agreement for the diagnosis of HP is modest (Walsh et al., 2016; Zaizen et al., 2020). To sum up, the possibility of IPF misdiagnosed as CHP based on GSE150910 dataset cannot be ruled out. Moreover, the additional public datasets of m6A regulators will be helpful to verify the model we constructed.

We found that the m6A score in cluster A was higher than that in cluster B. In addition, we conducted WGCNA method to divide modules based on the treatment samples. KEGG pathways of the genes in blue modification pattern suggest that TNF signaling pathway and ECM-receptor interaction are possible features of the evolution of fibrosis.

## Conclusion

In conclusion, this study revealed the regulation of m6A methylation factors in pulmonary fibrosis. The comprehensive evaluation of two m6A modification patterns may contribute to distinguishing non-fibrotic CHP and IPF, providing guidance to new immunotherapy strategies for the patients. Going forward, we will further gather clinical specimens to establish an experimental model of pulmonary fibrosis and validate the findings of this study.

## Data Availability

The original contributions presented in the study are included in the article/Supplementary Material; further inquiries can be directed to the corresponding authors.
