# Corrigendum: Relevance of RNA N6-methyladenosine regulators for pulmonary fibrosis: Implications for chronic hypersensitivity pneumonitis and idiopathic pulmonary fibrosis

**DOI:** 10.3389/fgene.2022.1056103

**Published:** 2022-10-18

**Authors:** Yiyi Zhou, Chen Fang, Qinying Sun, Yuchao Dong

**Affiliations:** Department of Respiratory and Critical Care Medicine, Changhai Hospital, Second Military Medical University, Shanghai, China

**Keywords:** chronic hypersensitivity pneumonitis, idiopathic pulmonary fibrosis, M6A, immune microenvironment, consensus clustering

In the published article, there was an error in the Funding statement where the funding number was incorrectly filled in. The correct Funding statement appears below:

“This project was sponsored by funding from the Natural Science Foundation of Shanghai (19ZR1455800).”

The authors apologize for this error and state that this does not change the scientific conclusions of the article in any way. The original article has been updated.

